# *Shigella* Bacteremia in a Patient with Visceral Leishmaniasis

**DOI:** 10.1155/2013/920729

**Published:** 2013-08-19

**Authors:** Mengistu Endris, Rezika Mohammed, Yegnasew Takele, Desalegn Woldeyohannes, Moges Tiruneh, Ermias Diro

**Affiliations:** ^1^School of Biomedical and Laboratory Sciences, University of Gondar, P.O. Box 196, Gondar, Ethiopia; ^2^Leishmanaisis Research and Treatment Center, University of Gondar, P.O. Box 196, Gondar, Ethiopia; ^3^Department of Internal Medicine, University of Gondar, P.O. Box 196, Gondar, Ethiopia; ^4^Institute of Tropical Medicine, Antwerp, Belgium

## Abstract

Bacteremia due to *Shigella* is rare. A 26-year-old HIV-negative male presented with a persistent high-grade fever of two months duration to the Leishmaniasis Research and Treatment Center of University of Gondar Hospital. He was anorexic and had lost significant weight (from 76 to 57 kg in 4 months, BMI = 17.2 kg/m^2^). He also complained of headache, chills, and rigor. In the last one year, he was experiencing a few episodes of acute bloody diarrhea, the last episode being two months ago. Microscopy from splenic aspiration showed Leishman-Donovan bodies with parasite load of +3. The blood culture showed *Shigella* species, but the stool was culture negative. The isolate was sensitive to most tested antibiotic discs, sulfamethoxazole, ceftriaxone, gentamicin, tetracycline, and norfloxacilin, except ampicillin. Therefore, requesting blood culture for identifying unexpected type of organisms causing infections in patients with underlying diseases like visceral leishmaniasis should be encouraged.

## 1. Introduction

Bacteremia due to* Shigella *is rare. *Shigella* is a gram-negative, nonmotile facultative anaerobe that causes infection typically confined to the gastrointestinal tract [1]. The disease is mediated by enterotoxin and manifests with acute bloody diarrhea and fever often occurring in an outbreak due to contamination of water [2]. There are few reports of *Shigella* causing meningitis, osteomyelitis, and sepsis mostly in neonates, malnourished children, and immuno compromised hosts [3–8]. It is highly likely that such kind of infection be missed and associated with high risk of death.

*Shigella* rarely invades the bloodstream and results in septic shock. Macrophages not only fail to kill *Shigella *bacteria that they phagocytize, but also are killed by them [1]. Due to overlapping manifestations of sepsis, blood culture is not a routine examination in visceral leishmaniasis (VL) patients. To the best of our knowledge, *Shigella* bacteremia was not reported in patients with VL in Ethiopia. Here we report a case of *Shigella* bacteriemia in a patient with VL. 

## 2. Case Description

### 2.1. Clinical Presentation

 A 26-year-old male presented with a persistent high-grade fever of two months duration to the Leishmaniasis Research and Treatment Center of University of Gondar Hospital. He was anorexic and had lost significant weight (from 76 to 57 kg in 4 months). He also complained of headache, chills, and rigor.

In the last one year, he was experiencing some acute episodes of bloody diarrhea, the last episode being two months ago. He was treated with unspecified medications at a health center and then improved.

He lived in malaria and VL endemic areas and had suffered from repeated malaria attacks. Since the last eight years he had noticed left upper quadrant swelling. He has tinnitus, vertigo, and blurring of vision. He has no history of epistaxis or easy bleeding. He has no self or family history of diabetes mellitus. He has no cough or other respiratory symptoms.

On presentation he was acutely sick looking and febrile (39.2°C), tachycardic (112/minute), and tachypneic (28/minute). His blood pressure was 90/60 mmHg. His body mass index was 17.2 kg/m^2^. He had splenomegaly of 9 cm below the coastal margin. He had some palmer pallor but no edema.

His peripheral blood count showed pancytopenic pattern: total white blood cell count of 800 cells/*μ*L, hemoglobin of 8 gm/dL, and platelet of 51,000/*μ*L. His absolute neutrophil count was 432/*μ*L. Rk 39 rapid deep-stick serology test for leishmaniasis was positive. Microscopy from splenic aspiration showed Leishman-Donovan bodies of grade +3 parasite loads. His human immunodeficiency virus (HIV) serology test was negative. Urinalysis, liver enzymes, and bilirubin were all normal. There was no hemoparasite detected on blood-film examination. The stool had no intestinal helminthes using saline wet mount.

The patient was treated with intravenous ceftriaxone for 7 days and sodium stibogluconate (SSG) for 30 days. His vital signs stabilized after 7 days. The blood count started to improve starting from the first week of treatment and normalized at the end of the month. 

### 2.2. Bacteriology

Taking blood culture for VL patients was not routine. Blood culture was taken based on clinical indications for septic patients. As one of the common causes of mortality among VL patients was sepsis, blood culture was taken for this patient taking into account his presentation with systemic inflammatory response syndrome and severe neutropenia.

Ten mL of blood was drawn from the patient and inoculated on 45 mL brain heart infusion (BHI) following the standard procedure [9]. The culture was incubated at 37°C; after 4 days of incubation it showed positive sign. Then the sample was inoculated on blood agar plate and MacConkey agar plates. Gram-negative rod-shaped bacteria were seen under the microscope using Gram stain. It was then further tested by biochemical tests such as indole test, triple sugar iron agar, nitrate and simmon citrate, and lysine decarboxylase. The bacteria isolated was gram-negative, rod, nonmotile, and negative for citrate, indole, and urea. Neither lactose nor glucose was fermented by the bacteria. The laboratory result showed *Shigella* species.

The antimicrobial susceptibility of the isolate was done using the disc diffusion technique, and the result was interpreted according to Clinical and Laboratory Standards Institute [10]. The isolate was sensitive to all the tested antibiotic discs, sulfamethoxazole, ceftriaxone, gentamicin, tetracycline, and norfloxacilin, except ampicillin. 

The stool sample was also collected from the patient and processed to find *Shigella* using Salmonella Shigella (SS) agar. There was no *Shigella* grown on the stool culture. 

## 3. Discussion

Bacteremia due to shigellosis is clinically unexpected. As this patient was having VL that manifests typically with chronic fever, weight loss, pancytopenia, and its complications like other bacterial superinfections and bleeding, it is very difficult to know whether the *Shigella* bacteremia had unique clinical manifestations or not. 

Majority of the *Shigella* bacteremia cases occurred in children and adults with an underlying disease, such as diabetes, leukemia, sickle cell disease, malignancy, cirrhosis, immunosuppressive therapy, and HIV infection [3–8].

This patient used to have recurrent diarrhea despite repeated treatments done for him. Though the appropriateness of the treatments given cannot be proven, frequent relapse of the disease potentiates the failure of his immunity to clear the infection. *Shigella* infection represents an interesting paradigm of imbalance of the host immune mechanism to regulate infection and of bacterial strategies developed to escape killing by host immune cells that are equipped with a large repertoire of antimicrobial weapons to control microbial infection [11].

Leishmaniasis is associated with many immunological disorders including depression of cell-mediated immunity to Leishmania or other non related antigens [12]. In patient with VL the skin, respiratory tract, and middle ear were the most common sites of infections, and *Pseudomonas aeroginosa* and *Staphylococcus aureus* were the most common etiologic agents [13]. 

The underlying disease in our case described here was VL. The patient was having severe leucopenia and malnutrition. Immune suppressive and debilitating conditions like VL may allow *Shigella* to easily pass the intestinal epithelia and get into the blood stream. 

The clinical significance of *Shigella* bacteremia is not clear [14]. Higher case fatality rate among bacteremic *Shigella* infection was reported than the nonbacteremic ones. However, this may be difficult to conclude as the high mortality could be from the severe underlying disease than the *Shigella* bacteremia. In the review of records of 2018 patients with shigellosis in a hospital in Bangladish between 1976 and 1983, it was found that severe complications occurred more frequently after *Shigella* bacteremia than in controls [15]. Luckily our patient was not having any of the complications even if he has an underlying VL. The review also showed similar median duration of illness between the *Shigella* bacteremia patients and the controls (five and four days), but in our patient since the patient was having VL which is characterized by chronic symptoms it is difficult to know the exact duration of symptoms of *Shigella* bacteremia. Mostly patients reported with *Shigella* bacteremia had positive result on stool culture as well; however, that was not true in our case. 

The presence of *Shigella* bacteremia in a VL patient indicates that VL is one of the immune suppressive conditions that can predispose for bacteremia due to atypical pathogens. Therefore, requesting blood culture to identify unexpected type of organisms causing infections in patients with underlying diseases like VL should be encouraged. Obviously, this type of evidence-based management will improve the treatment outcome of patients.
